# Assessing the inclusion of frailty scores in either the methodology or results of clinical intervention trials of heart failure: A scoping review

**DOI:** 10.1371/journal.pone.0358624

**Published:** 2026-11-12

**Authors:** Murhaf Al-Shalabi, Nafeesa Abdul Nazar, Martyn Patel

**Affiliations:** 1 Ipswich Hospital, East Suffolk and North Essex NHS Foundation Trust, Ipswich, United Kingdom; 2 Norwich Medical School, University of East Anglia, Norwich, United Kingdom; 3 Norfolk and Norwich University Hospital, Norwich, United Kingdom; PLOS: Public Library of Science, UNITED KINGDOM OF GREAT BRITAIN AND NORTHERN IRELAND

## Abstract

Chronic heart failure is highly prevalent in older adults, in whom frailty is common and associated with poorer outcomes. Despite advances in guideline-directed medical therapy, concerns regarding tolerability and polypharmacy contribute to under-utilization in frail patients. This scoping review aimed to map the existing evidence on how clinical interventional trials of heart failure management have incorporated frailty, specifically, whether trials have (i) stratified outcomes according to frailty status and/or (ii) adjusted treatment strategies based on frailty. We searched MEDLINE, Ovid Embase, Cochrane CENTRAL and ClinicalTrials.gov for interventional trials of heart failure published up to August 2025 that incorporated frailty using validated assessment tools. Eligible studies stratified outcomes by frailty status and/or adjusted interventions based on frailty. Data were extracted and a narrative synthesis was conducted. Ten studies met the inclusion criteria. Seven were post-hoc analyses of randomized controlled trials, two were prespecified analyses, and one was a randomized controlled trial. Two studies evaluated exercise-based interventions, one on implantable cardioverter-defibrillators, and the remainder on pharmacological-based therapies. Nine studies used the Rockwood cumulative deficit approach to measure frailty, although the parameters used varied between studies. Available secondary analyses suggest preserved efficacy across frailty groups for most pharmacological interventions, whereas other modalities did not show convincing improvements in heart failure-specific outcomes among frail patients. Primary evidence of heart failure interventions incorporating frailty remains limited, with a large bulk of the evidence available being retrospective. Although several reported pharmacological interventions appear to have preserved efficacy across frailty levels, some of these therapies were not recommended in current guidelines, and heterogeneity in frailty measurement reduced the confidence of conclusions drawn. The gaps identified in this review highlight the need for further primary evidence that incorporates frailty, plus a standardized holistic method of measuring frailty in heart failure patients.

## Introduction

Chronic heart failure (HF) is highly prevalent in older populations and is associated with substantial morbidity and mortality. The prevalence is estimated to be over 10% for those aged over 70 years [1], and HF was a contributing cause for 45% of cardiovascular deaths in the United States in 2022 [2]. There are around 200,000 new diagnoses of HF every year in the United Kingdom [3]. Together, these figures underscore the significant and growing public health burden of HF, particularly in aging populations.

Over the past few decades, extensive research has evaluated the effects of both pharmacological and non-pharmacological interventions on outcomes in HF. Current guidelines recommend the initiation of the “four pillars of HF therapy” for patients with reduced or mildly reduced ejection fraction [4], with evidence showing significant mortality benefits when these therapies are initiated and appropriately up-titrated [5].

Despite these advances, many geriatricians question the applicability of these findings to frail older adults. Frailty is a multidimensional syndrome characterized by a state of increased vulnerability to stressors due to reduced physiological reserve (not to be considered synonymous with aging, multimorbidity, disability, or disease severity) [6]. Two main models currently exist for frailty, the Fried frailty phenotype and the Rockwood cumulative deficit model [6,7]. The Fried frailty phenotype conceptualizes frailty based on the presence of specific clinical features, including unintentional weight loss, exhaustion, low physical activity, slow gait speed, and reduced grip strength. In contrast, the Rockwood cumulative deficit approach describes frailty as the cumulative burden of multiple health deficits across clinical domains. These models highlight the complexity and multidimensional nature of frailty. Concerns have been raised regarding the safety and tolerability of intentional polypharmacy in frail patients, as well as the external validity of clinical trial findings in this population [8].

This scoping review aims to map the existing evidence on how clinical interventional trials of HF management have incorporated frailty. Specifically, we investigated whether trials have (i) stratified outcomes according to frailty status and/or (ii) adjusted treatment strategies based on frailty, in order to better understand how current evidence addresses the needs of this high-risk population.

## Materials and methods

The scoping review was conducted in accordance with the JBI methodology for scoping reviews [9]. The review title was registered with the Open Science Framework.

### Search strategy

We conducted a search of four databases, namely Ovid Embase, Medline, Cochrane CENTRAL, and clinicaltrials.gov, up to August 2025. The search strategy combined MeSH terms and free-text keywords relating to three core concepts: heart failure, frailty, and clinical interventional trials. These concepts were combined using Boolean operators (AND/OR), with database-specific adaptations applied where appropriate. An example of the search strategy used can be found in the scoping review protocol (S1 File). From this search strategy, we selected publications that report evidence of either clinical interventional outcomes by frailty in patients with HF, or outcomes of different interventional strategies for HF based on frailty. We restricted the results to studies published in English, as most studies are published in this language. The above search strategy located only published studies and excluded conference abstracts.

### Inclusion criteria

#### Participants.

Adult patients (Age ≥ 18) with a diagnosis of HF, including HF with preserved ejection fraction, mildly reduced ejection fraction, and reduced ejection fraction (HFpEF, HFmrEF and HFrEF respectively) and a range of frailty levels across the cohort.

#### Concept.

Whether the clinical interventional trial for HF differentiated the reporting of outcomes or the method of intervention based on frailty. Frailty must be measured by a validated assessment tool.

#### Context.

Clinical interventional trials performed in any healthcare setting.

### Types of sources

This scoping review considered both experimental and quasi-experimental study designs that involved a clinical intervention of HF including randomized controlled trials, non-randomized controlled trials, before and after studies and interrupted time-series studies.

### Study/Source of evidence selection

Following the search, all identified citations were collated and uploaded into a spreadsheet processing software and duplicates were removed. Following a pilot test, titles and abstracts were screened by two or more independent reviewers for assessment against the inclusion criteria for the review. Potentially relevant sources were retrieved in full, and the full text of selected citations were assessed in detail against the inclusion criteria by two or more independent reviewers. Reasons for exclusion of sources of evidence at full text that do not meet the inclusion criteria were recorded and reported in the scoping review. Any disagreements that arose between the reviewers at each stage of the selection process were resolved through discussion, or with an additional reviewer.

### Data extraction

Data were extracted from the final list of publications (that are available in full text) on the study characteristics, population details, frailty assessment tool used, intervention, methodology, outcomes reported, stratification by frailty, and any notable practice. Data were extracted using a customized spreadsheet. Any disagreements that arose between the reviewers were resolved through discussion with the senior author.

### Data analysis and presentation

Findings were presented in tabular and narrative synthesis form, mapping the extent and nature of frailty incorporation into HF trials. A narrative summary accompanied the tabulated results and described how the results relate to the review objectives and questions.

## Results

Ten articles were analyzed in this scoping review. The study inclusion process is presented in a PRISMA flow diagram (Fig 1) [10].

**Fig 1:**
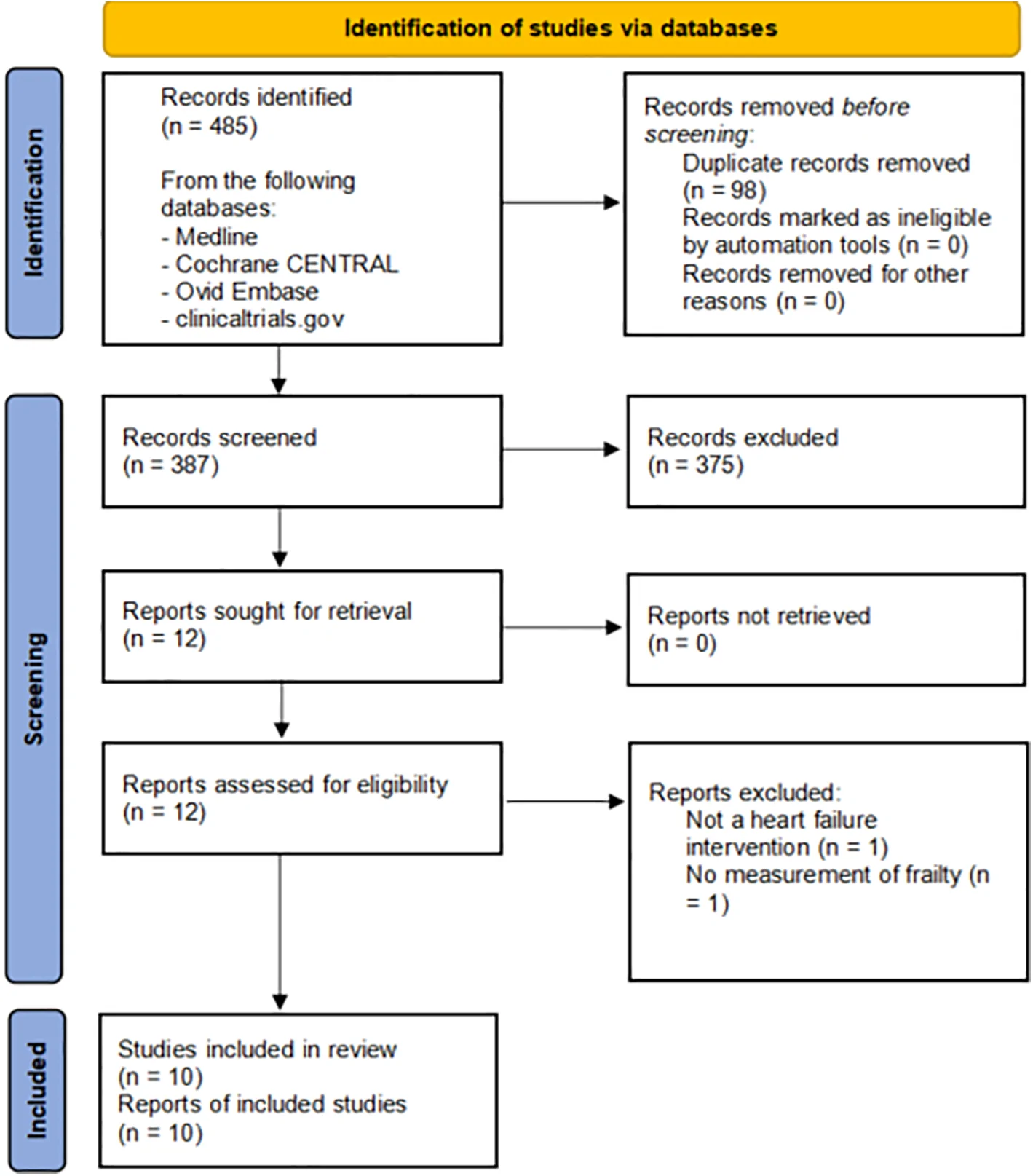
PRISMA flow diagram.

The characteristics of the ten included studies are shown in Table 1 [11-20].

**Table 1:** Study characteristics.

Reference	Study focus and patient population	Type of study	Country of origin of trial	Sample size	Frailty assessment tool	Key exclusion criteria
Butt et al. (2022a) [11]	Efficacy/tolerability of dapagliflozin in patients with HFpEF or HFmrEF according to frailty status	Prespecified quantitative analysis of double-blind RCT	Multi-national	6258	Rockwood cumulative deficit approach	T1DM, eGFR < 25, SBP < 95
Butt et al. (2022b) [12]	Efficacy/tolerability of dapagliflozin in patients with HFrEF according to frailty status	Post-hoc quantitative analysis of double-blind RCT	Multi-national	4339	Rockwood cumulative deficit approach	eGFR < 30, SBP < 95 or symptomatic hypotension, T1DM
Butt et al. (2022c) [13]	Efficacy of sacubitril/valsartan in patients with HFpEF according to frailty status	Post-hoc quantitative analysis of double-blind RCT	Multi-national	4795	Rockwood cumulative deficit approach	SBP < 110, eGFR < 30
Kitzman et al. (2021) [14]	Physical rehabilitation for older patients with acute decompensated HF	Randomised, attention-controlled, single-blind trial	USA	349	Fried’s frailty score	eGFR < 20, severe aortic valve stenosis
Sanders et al. (2018) [15]	Efficacy of spironolactone in patients with HFpEF according to frailty status	Post-hoc quantitative analysis of double-blind RCT	USA, Canada, Brazil, Argentina	1767	Rockwood cumulative deficit approach	AF with resting HR > 90, orthostatic hypotension, eGFR < 30
Pandey et al. (2022) [16]	Efficacy of aerobic exercise therapy on patients with HFrEF according to baseline frailty	Post-hoc quantitative analysis of non-blinded RCT	USA, Canada, France	2130	Rockwood cumulative deficit approach	Major co-morbidities that interfere with exercise training
Segar et al. (2024) [17]	Efficacy of ICD on patients with HFrEF according to baseline frailty	Post-hoc quantitative analysis of double-blind RCT	North America, Australia, New Zealand	1676	Rockwood cumulative deficit approach	No explicitly documented exclusion criteria found
Coats et al. (2023) [18]	Efficacy of empagliflozin on patients with HFpEF according to frailty status	Post-hoc quantitative analysis of double-blind RCT	Multi-national	5988	Rockwood cumulative deficit approach	Symptomatic hypotension, SBP < 100, eGFR < 20
Butt et al. (2025) [19]	Efficacy/safety of finerenone in patients with HFpEF or HFmrEF according to frailty status	Prespecified quantitative analysis of double-blind RCT	Multi-national	5952	Rockwood cumulative deficit approach	eGFR < 25, symptomatic hypotension and SBP < 90
Khan et al. (2022) [20]	Association of frailty with outcomes of optimal guideline-directed medical therapy in patients with HFrEF	Post-hoc quantitative analysis of non-blinded RCT	USA and Canada	879	Rockwood cumulative deficit approach	End stage renal disease (doesn’t specify eGFR cut off)

**Abbreviations:** RCT, Randomised controlled trial; T1DM, Type 1 Diabetes Mellitus; SBP, Systolic blood pressure; eGFR, estimated glomerular filtration rate; AF, atrial fibrilation; HF, heart failure; HFpEF, Heart failure with preserved ejection fraction; HFmrEF, Heart failure with mildly reduced ejection fraction; HFrEF, Heart failure with reduced ejection fraction; ICD, Implantable Cardioverter Defibrilator;

Seven out of the ten studies analyzed were post-hoc quantitative analyses of randomized controlled trials (RCTs) [12,13,15–18,20]. Two studies were prespecified quantitative analyses of double-blind RCTs [11,19]. One study was a RCT in itself with a sample size of 349 from one country (USA) [14]. The other studies analyzed were all based on multiple countries of origin with sample sizes upwards of 800. The Rockwood cumulative deficit approach for measuring frailty was used by nine out of ten studies (see Table 1) [7]. Fried’s frailty phenotype was used in one study [6,14]. Exclusion criteria were broadly consistent and aimed to limit confounding from comorbidities that may mimic or worsen HF symptoms, such as symptomatic anaemia, primary pulmonary hypertension, and recent acute myocardial infarction. Some notable exclusion criteria have been recorded in Table 1, highlighting the variation in eGFR and systolic blood pressure thresholds between studies.

Table 2 detail the PICO (population, intervention, comparator, outcome) information for these studies. Table 3 summarizes the outcomes and any notable comments on methodology. It also highlights whether these studies reported outcomes by frailty or adjusted the intervention based on frailty; no studies did both.

**Table 2:** Study PICO information.

Reference	Population	Intervention	Comparator	Outcome(s) measured
Butt et al. (2022a) [11]	HFpEF and HFmrEF patients	Dapagliflozin	Placebo	HF hospitalization, urgent HF visit, CV death, change in KCCQ scores, total HF events, all-cause mortality
Butt et al. (2022b) [12]	HFrEF patients	Dapagliflozin	Placebo	HF hospitalization, urgent HF visit, CV death, change in KCCQ scores, total HF events, all-cause mortality
Butt et al. (2022c) [13]	HFpEF patients	Sacubitril/Valsartan	Valsartan	Total HF hospitalizations, CV death, change in KCCQ scores, change in NYHA class, renal function decline, all-cause death
Kitzman et al. (2021) [14]	Older patients with acute HF	Physical rehabilitation programme	Usual care	SPPB (primary), all-cause rehospitalizations (split into non-CV, HF, other CV cause)
Sanders et al. (2018) [15]	HFpEF patients	Spironolactone	Placebo	CV death, hospitalization for HF, all-cause mortality
Pandey et al. (2022) [16]	HFrEF patients	Aerobic exercise training	Usual care	All cause hospitalization and mortality, CV hospitalization and mortality
Segar et al. (2024) [17]	HFrEF patients	ICD (single-lead, shock only)	Placebo	CV mortality, SCD, All cause hospitalization and mortality
Coats et al. (2023) [18]	HFpEF patients	Empagliflozin	Placebo	Time to CV death or HF hospitalization, change in eGFR, CV mortality and HF hospitalizations
Butt et al. (2025) [19]	HFpEF and HFmrEF patients	Finerenone	Placebo	CV death, total worsening HF events, change in NYHA class, change in KCCQ scores, change in eGFR, all cause mortality
Khan et al. (2022) [20]	HFrEF patients	Guideline-directed medical therapy	Usual care	Time to HF hospitalization or CV death, all-cause mortality, HF hospitalizations

**Abbreviations:** HFrEF, Heart failure with reduced ejection fraction; HFpEF, with preserved ejection fraction; HFmrEF, Heart failure with mildly reduced ejection fraction; HF, heart failure; CV, cardiovascular; SCD, Sudden cardiac death; ICD, Implantable Cardioverter Defibrilator KCCQ, Kansas City Cardiomyopathy Questionnaire; NYHA, New York Heart Association; eGFR, estimated glomerular filtration rate; SPPB, Short Physical Performance Battery;

**Table 3:** Study outcomes and frailty incorporation.

Reference	Incorporation of frailty	Summary of outcomes	Notable practice	Treatment-by-frailty interaction analysis (P_interaction_)
Butt et al. (2022a) [11]	Reported outcomes by frailty	Dapagliflozin improved outcomes with no increased adverse events regardless of frailty	None noted	Primary outcome: 0.4 Other outcomes: >=0.25
Butt et al. (2022b) [12]	Reported outcomes by frailty	Dapagliflozin improved outcomes with no increased adverse events regardless of frailty	None noted	No P_interaction_ values reported
Butt et al. (2022c) [13]	Reported outcomes by frailty	Sacubitril/Valsartan improved CV death and total HF hospitalizations more in frailer patients with no increase in adverse events	None noted	Primary outcome: 0.23 (0.002 if FI is continuous variable) Secondary outcomes >0.7
Kitzman et al. (2021) [14]	Reported outcomes by frailty	Improvement in SPPB regardless of frailty, no significant differences in secondary outcomes (not reported by frailty)	HF-related outcomes not reported by frailty	No analysis done
Sanders et al. (2018) [15]	Reported outcomes by frailty	No evidence of difference in treatment effect of spironolactone by frailty class.	None noted	All outcomes: 0.35
Pandey et al. (2022) [16]	Reported outcomes by frailty	Significant reduction in all-cause hospitalization and mortality regardless of frailty, non-significant reduction in CV hospitalization and mortality	None noted	Primary outcome: 0.04 All-cause hospitalization: 0.09 Other outcomes: > 0.1
Segar et al. (2024) [17]	Reported outcomes by frailty	Significant reduction in outcomes in low frailty patients but not in high frailty patients (except for SCD where reduction was found in both)	None noted	All-cause mortality: 0.047 CV mortality: 0.02 Other outcomes: > 0.1
Coats et al. (2023) [18]	Reported outcomes by frailty	Maintained clinical benefits regardless of frailty. Side effect event rate increased as frailty worsened.	None noted	Primary outcome: 0.097 HF hospitalizations: 0.077 eGFR decline: 0.159
Butt et al. (2025) [19]	Reported outcomes by frailty	Finerenone improved outcomes with no increased adverse events regardless of frailty	Data on muscle strength and functional capacity not collected	Primary outcome: 0.77 Secondary outcomes all > 0.1
Khan et al. (2022) [20]	Reported outcomes by frailty	Uptitrated GDMT associated with improved outcomes regardless of frailty. High frailty patients more likely to receive sub-optimal under-titrated GDMT	Introduced heterogeneity in intervention	No analysis done on measured outcomes (analysis instead done on optimization of GDMT)

**Abbreviations:** HF, heart failure; CV, cardiovascular; SCD, sudden cardiac death; GDMT, guideline-directed medical therapy; SPPB, Short Physical Performance Battery; FI, Frailty Index; eGFR, estimated glomerular filtration rate

Out of the 10 studies analyzed, 4 studies were conducted by the Butt et al. group who sought to determine the efficacy and safety of various pharmacological interventions in different frailty groups [11–13,19]. They found evidence to suggest that dapagliflozin is both effective and safe in frail patients of all classifications of HF, as well as sacubitril/valsartan for patients with HFpEF and finerenone for patients with HFpEF or HFmrEF. Two further studies also looked at the efficacy of a pharmacological intervention on patients with HFpEF according to frailty status, these interventions being spironolactone and empagliflozin compared to placebo [15,18]. Both studies found evidence to suggest that the efficacy of these interventions also didn’t change by frailty status. However, Coates et al. (2023)’s data showed a worsening side effect profile with increasing frailty [18], and there is limited evidence in the Sanders et al. (2018) study to show that spironolactone is still safe with increasing frailty [15].

Another two studies focused on exercise-based interventions on HF patients [14,16]. The RCT by Kitzman et al. (2021) suggested that a physical rehabilitation programme in patients with acute decompensated HF improved the short physical performance battery score (SPPB) regardless of frailty status. However, no significant difference in more HF-specific outcomes were observed (rehospitalizations due to a cardiovascular cause) [14]. Only the SPPB outcomes were reported by frailty so it remains unclear as to whether there could be a difference in HF-specific outcomes when split by frailty status. The other exercise-based study suggested that aerobic exercise training in patients with HFrEF improved all-cause hospitalizations regardless of frailty status, but no statistically significant reduction in more HF-specific outcomes [16].

One study looked at a device-related intervention in patients with HFrEF, finding evidence to suggest that, apart from sudden cardiac death, therapy via implantable cardioverter defibrillator (ICD) showed no significant improvement in outcomes in patients with a higher frailty burden compared to placebo [17]. The final study analyzed “guideline-directed medical therapy” (GDMT) where the goal was to suppress the NTproBNP concentration to <1000 [20]. The target population were patients with HFrEF and the comparator was usual care. The exact way in which GDMT was implemented was up to the clinician’s discretion, as long as the NTproBNP target was achieved. This meant that heterogeneity was introduced to the study as a consequence of the study’s design. Moreover, it was found that clinicians were more likely to under-titrate or underutilized GDMT in patients that were more frail, incidentally adjusting the intervention by frailty despite not being explicitly incorporated into the trial’s design. There was also evidence to suggest that GDMT improved outcomes in all frailty classes compared to usual care [20].

### Narrative synthesis

We conducted a narrative synthesis approach across the included studies, consistent themes emerged that highlight important directions for future research [21].

#### Frailty measurement.

The majority of studies (nine out of ten) analyzed in this review chose the Rockwood cumulative deficit approach for frailty measurement [7]. When analyzing the parameters used, differences can be seen in: the number of components making up the frailty index, the components themselves, the number of components in each sub-section (e.g., vitals, co-morbidities, etc.), the weighting of the scoring for each component, the number of available scoring options in each component (mainly seen in the quality of life subsections) and the inclusion of the Kansas City Cardiomyopathy Questionnaire [22]. Please refer to the supplementary spreadsheet comparing the individual components of the frailty indices used by each included study (S4 File). Therefore, despite the majority of these studies basing the methodology of their frailty measurement from the same approach, the subsequent implementation of the approach has introduced heterogeneity. Subsequently, there is a large degree of heterogeneity introduced in how frailty is measured despite using the same general underpinning theory of frailty. While this approach comprehensively captures frailty, the diversity in how the approach is implemented limits direct comparability across studies and introduces potential bias. A standardized frailty assessment in HF research could be explored in the future to eliminate possible confounding factors that could arise from this heterogeneity and enhance consistency in future studies.

#### Incorporation of frailty in study designs.

All studies analyzed in this review incorporated frailty via outcome reporting as opposed to intentional intervention adjustment by frailty. Reporting outcomes by frailty was felt to be an effective choice for stratifying the effectiveness and safety of an intervention across different patient cohorts [11-20]. Being able to determine which subset of patients may benefit most from an intervention is currently something that clinicians are very interested in investigating, given the increase in prevalence of multi-morbid, frail, and increasingly older patients [23]. There were no explicit uses of a methodology centered around the alteration of intervention based on frailty of participants from the studies analyzed, although the study by Khan et al. (2022) introduced heterogeneity into the intervention by giving clinicians the freedom for how GDMT was implemented as long as the NTproBNP level was suppressed to <1000 [20]. This method of incorporating frailty has it’s own attractions, the strengths of which have been explained in more detail in the Discussion section, but in brief include the ability to compare treatment regimens at different stages of frailty – for example as a way to combat polypharmacy by investigating the possibility of reducing the number of medication at more advanced stages of frailty. As the heterogeneity found in Khan et al.’s study was not by design, it’s difficult to use this to draw any conclusions on the effect of the differences in GDMT implementation on the measured outcomes.

#### Effects of frailty on outcomes.

All included studies demonstrated that increasing frailty burden was associated with worse primary and secondary outcomes regardless of the trial arm [11-20]. When combining this with the findings of several pharmacological-based studies analyzed in this review showing that treatment efficacy was preserved across frailty groups, it reinforces the importance of optimizing HF treatment in this population group. This is because although relative risk reduction may be similar across frailty groups, the higher event rates in frail patients mean that the absolute risk reduction would be larger. An example of this can be illustrated using data from the Butt et al. study on sacubitril/valsartan in HFpEF patients, where the sample sizes from the “not frail” group and the “more frail” group are relatively similar [13]. The relative reduction of primary outcome and urgent HF visits for patients in the “not frail” group was 5.11%, whereas the relative reduction for the same parameter in the “more frail” group was 7.06%. This translates to an absolute reduction of approximately 11 events per 1000 patients in the “not frail” group, and 36 events per 1000 patients in the “more frail group”. Therefore, for a difference of relative reduction of under 2%, there was an improvement in absolute reduction of more than 2.5% for the “more frail” patients. Note that these calculations are derived estimates based on the reported event rates, and are intended to illustrate the potential clinical impact of optimizing the treatment of high-risk populations.

#### Choice of intervention modality.

Three main interventional modalities were analyzed in this study. Seven out of the ten studies fell under the pharmacological modality [11–13,15,18–20]. Two studies looked at exercise-based interventions [14,16]. One study looked at device based intervention [17].

Out of the seven studies that looked at pharmacological interventions, six of these assessed the efficacy and safety of individual medications [11–13,15,18,19]. The Butt et al. group suggested that finerenone, sacubitril/valsartan and dapagliflozin have not shown to have reduced benefits or worsening side effect profile in HF related outcomes as frailty burden worsened [11,13,19]. Note that these studies were done on patients with HFpEF, where currently finerenone and sacubitril/valsartan are not recommended in latest guidelines [1,4]. They also suggested the same for dapagliflozin in patients with HFrEF [12]. Empagliflozin and spironolactone were also investigated in patients with HFpEF, where once again spironolactone is not currently recommended in latest guidelines [1,4,15,18]. It was still suggested that the efficacy of both these medications did not worsen as frailty increased [15,18].

The remaining study that investigated pharmacological intervention looked at GDMT rather than individual medications [20]. This suggested that GDMT had good efficacy regardless of frailty status, but frail patients were more likely to have sub-optimal medical management due to clinician concerns of safety of these medications. This highlights the main issue with having limited evidence in this domain, and as such further exploration is necessary – especially in medications that are currently recommended in latest guidelines for the appropriate HF classification [1,4].

Exercise-based interventions appeared to have improved functional capacity but had limited impact on HF-related outcomes such as hospitalization rates or cardiovascular mortality [14,16]. However, as there were only two studies on this modality, one of which did not report HF related outcomes based on frailty, further exploration on the effect of exercise-based intervention on HF related outcomes in frail patients may be helpful [14] Due to the notable improvement in functional capacity, future research in the possibility of adjunct therapy may also be beneficial [14,16].

There was very limited evidence for device-based interventions, but the one study in this review that investigated this suggested that the efficacy of ICDs decreased in frail patients for addressing HF morbidity and mortality [17].

#### Evidence gaps identified.

In summary, our narrative synthesis highlights these key points for further research:

1. More interventional trials that incorporate frailty in some form, as well as exploring other ways of incorporating frailty in the study design
2. A standardized and holistic method of measuring frailty in patients with HF for ease of comparison between studies and elimination of possible bias
3. Focusing efforts on interventions currently recommended in latest guidelines
4. Investigating the efficacy of different modalities, and the possibility of adjunct use of exercise to pharmacological therapy.

## Discussion

HF is a major public health problem due to its high morbidity and mortality, as well as its high prevalence in the elderly [1-3]. Despite frailty being common in the elderly, this scoping review appears to demonstrate that primary evidence of HF intervention incorporating frailty is limited to date. This lack of evidence may lead to skepticism amongst geriatricians regarding the safety of emerging interventions, often involving deliberate polypharmacy, for their patient group. Evidence of this was demonstrated by Sze et al. (2021), where frail patients were less likely to receive optimal medical therapy [24]. Skepticism is compounded by conflicting literature on the topic; despite the limited evidence, a systematic review by Nijskens et al. (2025) concluded that optimal guideline-directed treatment appeared to be safe in frail patients [25]. However, others challenge the ability of frailty measurement tools used in these studies to accurately reflect reality, hence questioning the validity of claims that guideline-directed treatment is safe in frail patients [26].

### Implications for clinical practice

Although current evidence suggests that treatment efficacy may be maintained across frailty categories, available data do not establish that frailty should currently modify the intensity of guideline-directed therapy itself. Frailty may represent both a marker of increased risk and a factor influencing physicians’ prescribing behaviour. Therefore, decisions to withhold guideline-directed therapy on the basis of frailty alone are not currently supported by the limited available evidence.

Beyond supporting future research, standardized frailty assessment tool for HF patients may already have immediate value in routine clinical care. Firstly, it offers a practical tool for risk stratification, helping clinicians identify patients at heightened vulnerability to adverse outcomes independent of, or in addition to, conventional HF severity markers. Secondly, a structured frailty assessment can support shared decision-making as communicating an individual's frailty status in objective terms may help patients and clinicians jointly weigh the anticipated benefits of guideline-directed therapy against the burden of polypharmacy and treatment intensity, particularly in those nearing the margins of trial eligibility criteria. Lastly, frailty assessment can serve as a screening step to identify patients who may benefit from multidimensional interventions that extend beyond pharmacological management alone, including cardiac rehabilitation, nutritional optimization, and comprehensive geriatric assessment. This multidimensional approach to frailty is not a novel concept in cardiology; Testa et al. previously demonstrated the feasibility and clinical value of multidimensional frailty evaluation in elderly outpatients with chronic HF, highlighting how frailty in this population reflects the interplay of multiple, potentially modifiable domains rather than a single physiological deficit [27]. Similarly, the concept of the “catabolic syndrome” described by Curcio et al. highlights the complex interplay between frailty, sarcopenia, malnutrition, inflammation and metabolic dysregulation in older adults, further supporting multidisciplinary management approaches [28].

### Future research

Helpfully, a handful of research groups have now performed post-hoc quantitative analyses of interventional trials to generate secondary evidence of the effect of frailty on outcomes [12,13,15–18,20]. Although this should be seen as a good starting point, ideally future clinical interventional trials of HF should aim to incorporate frailty in their study design, including a standardized robust approach to measure frailty. It’s important to note that only three of the six studies that reviewed the efficacy and safety of individual medication are relevant to current guidelines, those being the studies looking at dapagliflozin and empagliflozin [11,12,18]. This further emphasizes the lack of evidence in this domain, establishing the need for further studies on guideline-directed HF therapy in frail patients.

As highlighted in the narrative synthesis, an effective way of incorporating frailty in study design could be by reporting outcomes by frailty as it allows direct comparison between how different groups based on frailty levels were affected by the intervention – whether it’s by comparing efficacy, safety, etc. However, alternative approaches could also be explored, such as tailoring the intervention based on frailty. For example, it’s theoretically possible to use this study design to compare treatment with the four pillars of HF therapy in non-frail patients with HFrEF vs a different regimen of medical therapy involving less intensive medication for frail patients with HFrEF. This approach could provide valuable evidence to assess whether a different medical regimen could provide similar or even improved outcomes in efficacy and safety in frail patients whilst simultaneously combating polypharmacy that leads to adverse health outcomes and increased side effects – a recognized public health issue [29]. Frailty incorporation in study design can be done in multiple ways depending on the question that the study aims to answer, and as frailty in HF intervention is a relatively under-explored topic, it may be helpful to explore different ways of incorporating frailty to provide holistic evidence to aid clinicians in decision making.

We’ve discussed the benefits of incorporating frailty in HF interventional studies and how it will impact health care of this patient group. To derive maximum relevant information from clinical trials, the trial population should ideally match the age and frailty profile of the clinician’s workload. However, due to the extensive exclusion criteria in these studies, the applicability of the findings may be restricted to a narrower HF population, and unintentionally exclude those with increased frailty burden who are more likely to be hypotensive or suffer from orthostatic hypotension [30]. For example, in the post-hoc quantitative analysis of the PARAGON-HF trial done by Butt et al., the PARAGON-HF trial itself excluded patients with a systolic blood pressure of 110 – a relatively high cut-off in comparison to other trials, especially as latest guidelines suggest sacubitril/valsartan can be started at a lower dose in patients with a systolic blood pressure between 100 and 110 [13,31]. Hence, HF clinical interventional trials that choose to incorporate frailty in their study design will also need to carefully consider the exclusion criteria to ensure a substantial proportion of the target population do not get alienated from the relevance of the findings of the study.

### Limitations

This scoping review focused on clinical interventional trials that incorporated frailty in two stated methods – either by reporting outcomes or changing the intervention based on frailty. As a result, there may have been relevant evidence that was not reviewed in this study, such as case series, observational studies and those studies published in grey literature. Due to the focus of this scoping review being a mapping of the available evidence rather than a formal assessment of its strength or certainty, a formal critical appraisal of the evidence was not conducted. However, this limits interpretation of the quality of the evidence. Interpretation is further limited by the heterogeneity of frailty indices used in the included studies, as well as the dominance of secondary post-hoc analyses which are more susceptible to reduced power of interaction analyses, selection bias and residual confounding. Moreover, there is a potential for publication and language bias as unpublished studies and studies published in languages outside the search criteria may not have been captured, potentially limiting the comprehensiveness of the evidence base.

## Conclusion

This scoping review aimed to map the current evidence surrounding HF intervention in frail patients by exploring the extent to which clinical interventional trials have incorporated frailty in their study design. Current evidence suggests that several pharmacological interventions retain efficacy across frailty levels; however, confidence in these findings is limited by the scarcity of prospective studies and heterogeneity in frailty assessment methods. Future HF trials should incorporate standardized frailty measures prospectively and evaluate whether treatment strategies tailored to frailty can improve outcomes while minimizing treatment burden. In addition, we echo the calls of others to work towards a specific HF frailty score [32].

## Supporting information

S1 File Scoping review protocol. https://osf.io/jh8gx/. (DOCX)

S2 File Full data from article screening process. (XLSX)

S3 File Scoping review PRISMA checklist. (DOCX)

S4 File Frailty index comparison spreadsheet. (XLSX)
