# Supplementary material for: Assessing the inclusion of frailty scores in either the methodology or results of clinical intervention trials of heart failure: A scoping review
Source: PLoS One. 2026 Nov 12;21(11):e0358624. doi: 10.1371/journal.pone.0358624 (PMC13759108; doi:10.1371/journal.pone.0358624)
Supplement: Supplementary file 1 [file pone.0358624.s001.docx]

# Assessing the inclusion of frailty scores in either the methodology or results of clinical intervention trials of heart failure: A scoping review protocol.

## Authors

**Murhaf Al-Shalabi ^1^ Martyn Patel ^2,3^**

1. Ipswich Hospital, East Suffolk and North Essex NHS Foundation Trust, Ipswich, UK.

2. Norfolk and Norwich University Hospital NHS Trust, Norwich, UK.

3. Norwich Medical School, University of East Anglia, Norwich, UK.

## Abstract

**Objective:**

To determine if there are any published clinical intervention trials of heart failure that incorporate frailty scoring either in their methodology of trial design, or reporting of trial outcomes.

**Introduction:**

The rising caseload of heart failure combined with frailty phenotype means that clinicians, particularly geriatricians wish to know if following maximal titration of heart failure medications is beneficial or harmful when analysed by frailty scores. This review sets out to scope the current evidence base, to determine if answers to this question already exist, and what gaps in research knowledge can be identified.

**Inclusion criteria:**

Clinical intervention trials of heart failure in adults, where the clinical interventional trial differentiates the reporting of outcomes or the method of intervention based on frailty. Frailty must be measured by a validated assessment tool. Study published before August 2025, available in English.

**Methods:**

We will conduct a structured review of databases to identify suitable studies for inclusion. We will create a data extraction table from these studies, and conduct a narrative synthesis is there is sufficient data to analyse.

**Keywords:**

Heart Failure, Frailty, Co-morbidity

## Introduction

Chronic heart failure (HF) is a leading cause of hospitalisation in individuals over 65 years of age and is associated with substantial morbidity and mortality. Approximately 50% of patients die within five years of diagnosis.

Over the past two decades, extensive research has evaluated the effects of pharmacological and non-pharmacological interventions on outcomes in HF. Current guidelines recommend the initiation of the "four pillars of heart failure therapy" for patients with reduced or mildly reduced ejection fraction, with evidence showing substantial mortality benefits when these therapies are initiated and uptitrated rapidly. [1]

However, concerns remain about the applicability of these findings to frail older adults. Frailty is a clinical state of increased vulnerability, characterised by reduced physiological reserve and resilience to stressors, and is commonly operationalised using validated tools such as the Fried frailty phenotype or the Clinical Frailty Scale. Many geriatricians question the safety and tolerability of intentional polypharmacy in frail patients, as well as the external validity of clinical trial findings in this population.

This scoping review seeks to map the existing evidence on how frailty has been incorporated into clinical interventional trials of HF management, including whether outcomes are stratified by frailty status and whether treatment strategies differ for frail versus non-frail patients.

## Review question

Are there any clinical interventional trials of heart failure management that

(a) report outcomes by frailty

and / or

(b) report outcomes when using different treatment strategies for frail patients vs non-frail.

## Inclusion criteria

**Participants**

Adult patients (Age ≥ 18) with a diagnosis of heart failure (including heart failure with preserved ejection fraction, moderately reduced ejection fraction and reduced ejection fraction, respectively mHFpEF, HFmrEF and HFrEF) and a range of frailty levels across the cohort.

**Concept**

Whether the clinical interventional trial differentiates the reporting of outcomes or the method of intervention based on frailty. Frailty must be measured by a validated assessment tool.

**Context**

Will include clinical interventional trials performed in any healthcare setting.

**Types of sources**

This scoping review will consider both experimental and quasi-experimental study designs that involve a clinical intervention of HF including randomised controlled trials, non-randomised controlled trials, before and after studies and interrupted time-series studies.

## Methods

The proposed scoping review will be conducted in accordance with the JBI methodology for scoping reviews. [2] The review title has been registered with the Open Science Framework. [3]

**Search strategy**

We will conduct a search of 4 databases, namely Ovid Embase, Medline, Cochrane CENTRAL, and clinicaltrials.gov, up to August 2025. For details of the search terms used, please refer to the appendix.

We will select from this search strategy those results that are publications that report evidence of either clinical interventional outcomes by frailty in patients with heart failure, or outcomes of different interventional strategies of heart failure based on frailty.

We will restrict to studies published in English, as most relevant studies are published in this language.

The above search strategy will aim to locate only published studies and will exclude conference abstracts.

**Study/Source of evidence selection**

Following the search, all identified citations will be collated and uploaded into a spreadsheet processing software and duplicates removed. [4]

Following a pilot test, titles and abstracts will then be screened by two or more independent reviewers for assessment against the inclusion criteria for the review.

Potentially relevant sources will be retrieved in full, and the full text of selected citations will be assessed in detail against the inclusion criteria by two or more independent reviewers.

Reasons for exclusion of sources of evidence at full text that do not meet the inclusion criteria will be recorded and reported in the scoping review.

Any disagreements that arise between the reviewers at each stage of the selection process will be resolved through discussion, or with an additional reviewer/s.

The results of the search and the study inclusion process will be reported in full in the final scoping review and presented in a PRISMA flow diagram. [5]

**Data extraction**

We will extract data from the final list of publications (that are available in full text) on the study characteristics, population details, frailty assessment tool/definition used, intervention, methodology, outcomes reported, stratification by frailty, and conflicts of interest where reported.

**Data analysis and presentation**

Findings will be presented in tabular and narrative synthesis form, mapping the extent and nature of frailty incorporation into HF trials. Descriptive statistics (counts, proportions) will be used where appropriate.

A narrative summary will accompany the tabulated results and will describe how the results relate to the review objectives and questions.

## Conflicts of Interest

The authors declare no conflicts of interest.

## Funding

This review is not externally funded.

## References

1) Mebazaa A, Davison B, Chioncel O, Cohen-Solal A, Diaz R, Filippatos G, et al. Safety, tolerability and efficacy of up-titration of guideline-directed medical therapies for acute heart failure (STRONG-HF): a multinational, open-label, randomised, trial. Lancet 2022;400:1938–52. https://doi.org/10.1016/S0140-6736(22)02076-1
2) Peters MD, Godfrey C, McInerney P, Khalil H, Larsen P, Marnie C, Pollock D, Tricco AC, Munn Z. Best practice guidance and reporting items for the development of scoping review protocols. JBI evidence synthesis. 2022 Apr 1;20(4):953-68.
3) Open Science Framework <https://osf.io/>
4) Kingsoft Office Software, 2024. WPS Spreadsheet, Available at: https://www.wps.com/office/spreadsheet/
5) Tricco AC, Lillie E, Zarin W et al. PRISMA extension for scoping reviews (PRISMA-ScR): Checklist and explanation. Ann Intern Med 2018;169, DOI: 10.7326/M18-0850.

## Appendices

Appendix I: Example Search Strategy

For MEDLINE (PubMed)

("heart failure"[MeSH Terms] OR "congestive heart failure"[Title/Abstract] OR "cardiac failure"[Title/Abstract]) AND (frail* OR sarcopenia) AND ("randomized controlled trial"[Publication Type] OR "clinical trial"[Publication Type] OR trial[Title/Abstract])
